# Serologic Screening for *Neospora caninum*, France

**DOI:** 10.3201/eid1506.081414

**Published:** 2009-06

**Authors:** Florence Robert-Gangneux, Frédéric Klein

**Affiliations:** Université Rennes 1, Rennes, France (F. Robert-Gangneux); Laboratoire Départemental de l’Orne, Alençon, France (F. Klein)

**Keywords:** Parasites, Neospora caninum, dogs, humans, Toxoplasma, France, letter

**To the Editor:** In the June 2008 issue of Emerging Infectious Diseases, McCann et al. (*1*) reported on serologic screening for *Neospora caninum* antibodies and reported a lack of serologic evidence for *Neospora* infection in humans in England, where prevalence of infection with the closely related parasite *Toxoplasma gondii* also is low. Only limited data are available on human exposure to *Neospora*. We investigated the seroprevalence of *N. caninum* in humans in France, where *Toxoplasma* spp. seroprevalence is high.

Our study comprised 500 serum samples from healthy women, followed at the Cochin–Port Royal University Hospital in 1997 within the framework of toxoplasmosis surveillance during pregnancy, and 400 serum samples from HIV-infected patients. All serum samples were submitted to anti-*Toxoplasma* antibody testing by using indirect immunofluorescence (IIF; Toxo-spot IFI; bioMérieux, Marcy l’Etoile, France) and ELISA (Platelia Toxo IgG and IgM; BioRad, Hercules, CA, USA). An in-house microplate IIF test previously validated in cattle was used for simultaneous detection of anti-*Neospora* and anti-*Toxoplasma* immunoglobulin (Ig) G on the same microplate. All samples were screened at dilutions of 1:20 and 1:80, as is usually done in anti-*Toxoplasma* IIF assays in humans. Correlation between the anti-*Toxoplasma* IIF commercial test and in-house IIF was excellent (kappa coefficient = 0.98) and allowed us to compare the antibody titers against both parasites. Forty (8%) samples from immunocompetent persons and 21 (4%) from immunocompromised persons yielded a weak fluorescence when diluted 1:20. All but 4 had significant titers of anti-*Toxoplasma* IgG (>200 IU/mL in 77% of cases), which suggests low-level cross-reactions. Whereas titers of >200 and >320 are considered sufficient to diagnose neosporosis in dogs and cattle, respectively (*2*), positivity threshold was difficult to resolve in the absence of a positive human control. We decided on a positivity threshold of 1:80, which is similar to the threshold defined by others in further studies using an indirect fluorescence antibody test in humans (*3*,*4*). None of the 500 samples from immunocompetent persons were positive for *Neospora* antibodies when assessed at a dilution of 1:80. Within the group of immunocompromised persons, 3 were positive for *Neospora* antibodies at a titer of 80, and 1 was positive at a titer of 160. Three of these 4 HIV-infected patients had high titers of anti-*Toxoplasma* IgG (>2,000 IU/mL), suggesting *Toxoplasma* serologic reactivation. We found no evidence of *Neospora* infection or exposure in immunocompetent persons but could not exclude possible *Neospora* infection associated with *Toxoplasma* infection or reactivation in immunocompromised persons.

Taken together, our data agree with data from other studies conducted in European countries (*1*,*5*), which suggest that neosporosis in healthy humans is unlikely. However, the *Neospora* spp. seropositivity of some HIV-infected patients, although weak compared with the level of seropositivity in cattle or dogs, could suggest circulation of the parasite within immunocompromised hosts, a hypothesis supported by Lobato et al. (*3*). However, our observation of a strong serologic reactivation against *T. gondii* in 3 of 4 patients with anti-*Neospora* titers >80 mostly favors cross-reactivity involving homologous antigens of both parasites and nonspecific antibody binding from polyclonal stimulation of the immune system. Finally, one should keep in mind that the positive predictive value of a serologic test used in screening in low-prevalence populations is low. Large-scale studies are needed to more precisely determine the potential role of this parasite in immunodeficient humans and to isolate the parasite or detect *Neospora* DNA in such patients.
